# Supplementary material for: Kinetic properties of optogenetic site-specific DNA recombination by LiCre-loxP
Source: Biol Open. 2026 May 14;15(5):bio062381. doi: 10.1242/bio.062381 (PMC13225215; doi:10.1242/bio.062381)
Supplement: Supplementary information [file biolopen-15-062381-s1.pdf]

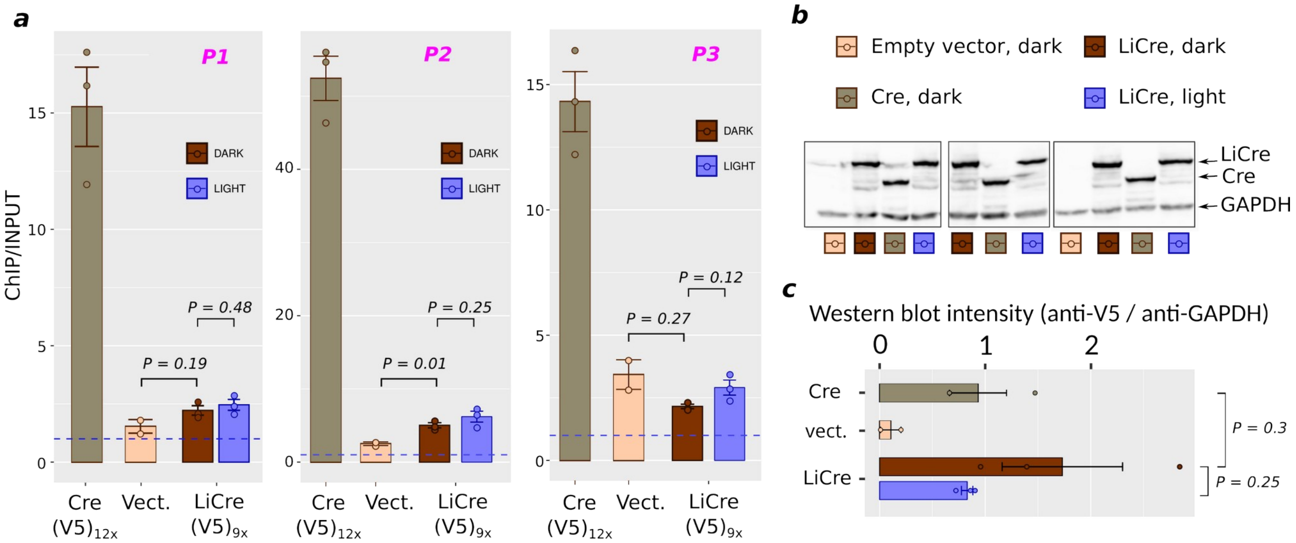

Fig. S1. Initial ChIP analysis of LiCre:DNA binding *in vivo*. **a**) ChIP-qPCR quantifications. Every dot corresponds to one biological replicate (independent culture inoculated from an independent transformant of Cre-$(V5)_{12x}$ (pGY607) or LiCre-$(V5)_{9x}$ (pGY605) expression plasmid. Values correspond to enrichment by ChIP (relative to its input), normalized by the signals obtained on three unrelated loci of the genome (see Methods). *P* values: Welch *t*-test. Error bars: mean +/- s.e.m. Strain used: GY2416. Design and probes are as shown on Figure 1a-c. **b**) Western blot analysis of whole-cell protein extracts. Proteins were extracted from the same population of cells as in a) (one biological replicate per lane). **c**) Quantification of LiCre, Cre and GAPDH immunoblot shown in e. *P* values: Welch t-test. In this experiment, unequal numbers of V5 epitopes were used for Cre and LiCre. Note that another ChIP experiment, entirely independent from this one and where equal numbers of epitopes were used, is shown in Fig. 1.

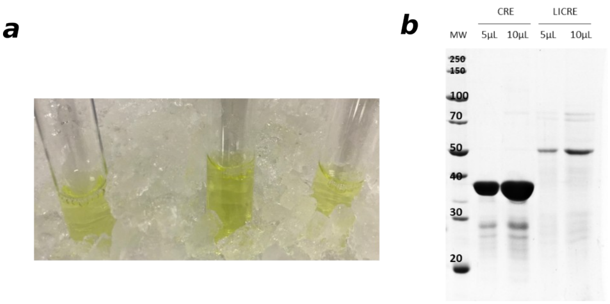

Fig. S2. a) Glass tubes containing eluted fractions of recombinant LiCre protein purified from *E. coli*. **b)** SDS-PAGE analysis of purified recombinant Cre and LiCre proteins.

Biology Open • Supplementary information

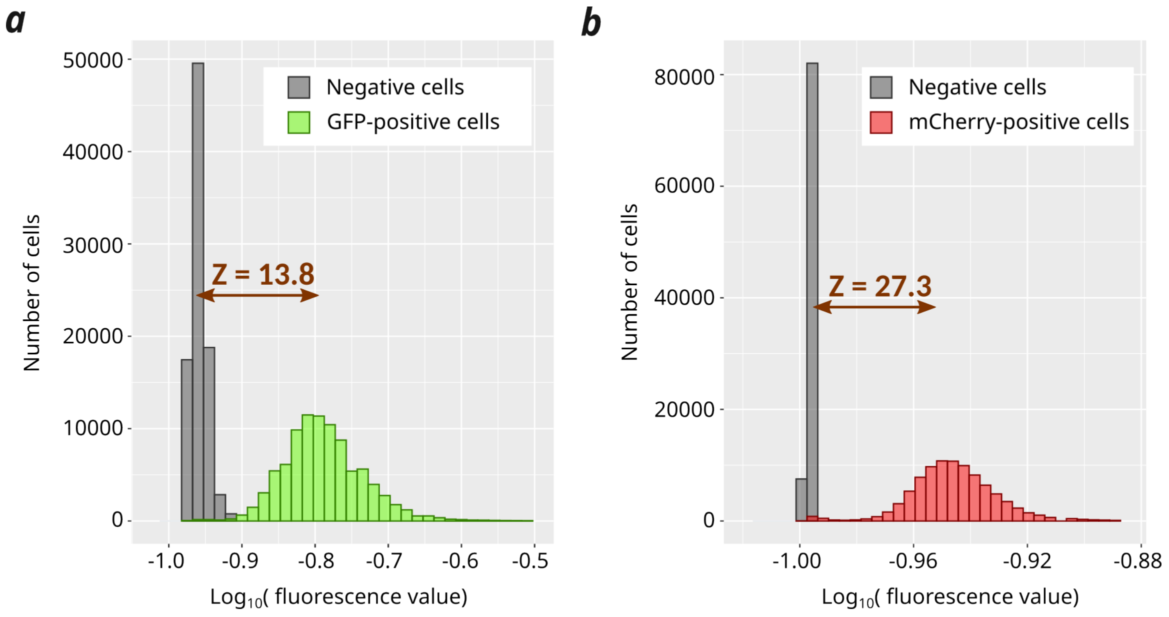

Fig. S3. Signal/noise ratio of the two yeast reporters. From dataset 8 of Supplementary Table S5. Strain used: GY2214. Negative cells correspond to cells transformed with an empty vector. Positive cells correspond to cells transformed with a plasmid for Cre expression (pGY502), which was induced during an overnight culture (achieving ~100% recombination). The distributions of fluorescent signals measured by flow-cytometry are shown for the GFP channel (panel **a**) and for the mCherry channel (panel **b**); for samples containing only negative cells (grey histogram) or only positive cells (colored histogram). *Z*, Z-score quantifying the distance between the mean fluorescent signal of positive cells and the overall signal of negative cells, computed as $Z = (\langle x\rangle_{pos} - \langle x\rangle_{neg}) / \sigma_{neg}$, where $\langle x\rangle_{pos}$ and $\langle x\rangle_{neg}$ are the mean signal of positive and negative cells, respectively, and σ_{neg} is the standard deviation of the signal among negative cells.

Biology Open • Supplementary information

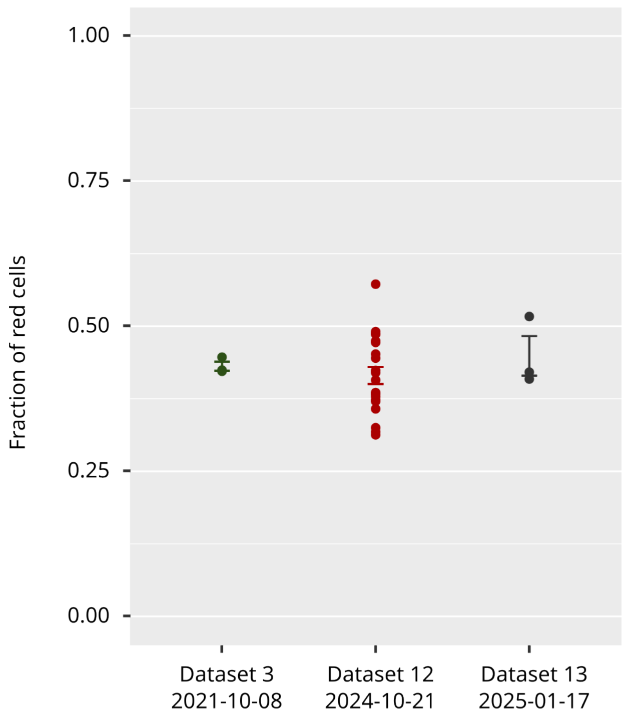

Fig. S4. Variability across experiments of LiCre-mediated recombination efficiencies after controlling for temperature. The same experimental conditions were used in three totally independent datasets produced at the indicated dates (as listed in Supplementary Table S5): strain GY2517, period of 10 sec, duty cycle of 10%, illumination duration of 60 min, intensity 35 mW/ cm2, controlled temperature of 30°C, LED box device. Each dot represents one biological replicate (independent culture). Bars: mean +/- s.e.m.

Table S1. List of plasmids used in this study.

ID	Name	System	Type	Description	Reference
pGY44	pRS314	yeast	Centromeric	Empty vectorBrachmann et al. 1998	Brachmann et al. 1998
pGY466	Pmet17-LiCre	yeast	Centromeric	LiCre expression under Pmet17 promoter	Duplus-Bottin et al. 2021
pGY472	pGY472	yeast	Integrative	HO-L:Ptef-loxP-KlLEU2-Tadh1-loxP-mCherry-Tcyc1:HO-R	Duplus-Bottin et al. 2021
pGY502	Pmet17-Cre	yeast	Centromeric	Cre expression under Pmet17 promoter	Duplus-Bottin et al. 2021
pGY537	pGY537	yeast	Integrative	pISLys2:Ptef-loxP-KlLEU2-Tadh1-loxP-GFP-URA3	Duplus-Bottin et al. 2021
pGY605	Pmet17-LiCre-(V5)9x	yeast	Centromeric	LiCre-V5 expression under Pmet17 promoter	This work
pGY607	Pmet17-Cre-(V5)12x	yeast	Centromeric	Cre-V5 expression under Pmet17 promoter	This work
pGY611	pET-LiCre	E. coli	IPTG expression	For production of recombinant LiCre in *E. coli*	This work
pGY618	pGY618	yeast	Integrative	pISLys2:Ptef-loxP-KlLEU2-Tadh1-loxP-mCherry-URA3	This work
pGY621	pGY621	yeast	Integrative	pISLys2:Tadh1-loxP-mCherry-Tcyc1-URA3	This work
pGY709	pET-Cre	E. coli	IPTG expression	For production of recombinant Cre in *E. coli*	This work
pGY775	pLiCre-Q513L	yeast	Centromeric	LiCre-Q513L expression under Pmet17 promoter	This work
pGY776	pLiCre-V416L	yeast	Centromeric	LiCre-V416L expression under Pmet17 promoter	This work
pGY777	pLiCre-F494I,L496S	yeast	Centromeric	LiCre-F494I,L496S expression under Pmet17 promoter	This work
pGY778	pLiCre-V416T	yeast	Centromeric	LiCre-V416T expression under Pmet17 promoter	This work
pGY779	pLiCre-N425Q,I427V	yeast	Centromeric	LiCre-N425Q,I427V expression under Pmet17 promoter	This work
pGY780	pLiCre-T418S	yeast	Centromeric	LiCre-T418S expression under Pmet17 promoter	This work
pGY781	pMet-LiCre-FMN1	yeast	Centromeric	LiCre expression under Pmet17 promoter and FMN1 expression under Padh1 promoter	This work
pGY820	Pmet17-Cre-(V5)9x	yeast	Centromeric	Cre-V5 expression under Pmet17 promoter	This work

Biology Open • Supplementary information

Table S2. List of yeast strains used in this study.

ID	Background	Genotype(i)	Source
OAy470	W303	*MATa ade2-1 his3-11,15 leu2-3,112 trp1-1 ura3-1 bar1::hisG*	Aparicio et al. 1997
GY855	S288c (BY)	*MATa leu2Δ0 trp1Δ63 ura3Δ0*	Duplus-Bottin et al. 2021
GY1761	S288c (BY)	*MATb his3Δ200 leu2Δ1 trp1Δ63 ura3Δ0 hoΔ::loxKlLEU2loxGFP*	Duplus-Bottin et al. 2021
GY2214	S288c (BY)	*MATa/MATb ADE2/ade2Δ::hisG his3Δ200/HIS3 leu2Δ0/leu2Δ1 LYS2/lys2Δ::loxKlLEU2loxGFP trp1Δ63/trp1Δ63 ura3Δ0/ura3Δ0 HO/hoΔ::loxKlLEU2loxmCherry*	Duplus-Bottin et al. 2021
GY2416	S288c (BY)	*MATa leu2Δ0 lys2Δ::Tadh1-loxP-mCherry trp1Δ63 ura3Δ0*	This work
GY2450	S288c (BY)	*MATa leu2Δ0 lys2Δ::loxKlLEU2lox-mCherry trp1Δ63 ura3Δ0*	This work
GY2517	S288c (BY)	*MATa/MATb his3D200/HIS3 leu2D0/leu2D0 LYS2/lys2Δ::loxKlLEU2loxmCherry trp1Δ63/trp1Δ63 ura3Δ0/ura3Δ0 HO/hoΔ::loxKlLEU2loxGFP*	This work
GY2752	W303	*MATa ADE2 his3-11,15 leu2-3,112 trp1-1 ura3-1 bar1::hisG*	This work
GY2753	W303	*MATa ADE2 his3-11,15 leu2-3,112 trp1-1 ura3-1 hoΔ::loxKlLEU2LoxmCherry bar1::hisG*	This work
GY2758	W303	*MATa ADE2. his3-11,15 leu2-3,112 trp1-1 ura3-1 bar1::hisG INO1:syn-mCherry-KlURA3 [ ]( )*	This work

(i): *MATb* corresponds to *MATα (alpha)*

Biology Open • Supplementary information

Table S3. List of oligonucleotides used in this study.

ID	Sequence (5' to 3')
1B36	TGCTGAGTTTTTGCGCATCAAT
1B37	TTTTGGTGCACGTTCGCT
1E10	CGTGAATAACCCAAATAACTGG
1E11	GCATACTTTTTATCCTTCACCG
1H97	GGGGTGACAATGTCTTGGCAAA
1H98	ACATCCGGAACAGCCTAATTCG
1R69	TTATCGATGCATGCCTGCAGGT
1R70	TTGACAGCCTTGGCGATAGCAT
1R71	AAAGTCCACATGGAGGGTTCAGTC
1R72	CGCCTTTGGTCACCTTCAATTTGG
1R73	TTTGAAGACGGTGGGGTTGTAACG
1R74	GCATGACAGGACCATCAGAAGGAA
1Y68	biotin-<br>CGGAATTCACAACTTCGTATAATGTATGCTATACGAAGTTATGGATCCGC
1Y69	GCGGATCCATAACTTCGTATAGCATACATTATACGAAGTTGTGAATTCCG
1Y70	biotin-CGGAATTCAGATCTATAACTTCGTATAATGTGTCTAGAGGATCCGC
1Y71	GCGGATCCTCTAGACACATTATACGAAGTTATAGATCTGAATTCCG
1Y72	biotin-<br>CGGAATTCAGATCTACATATGTGATATCTAAGCTTATCTAGAGGATCCGC
1Y73	GCGGATCCTCTAGATAAGCTTAGATATCACATATGTAGATCTGAATTCCG
1B36	TGCTGAGTTTTTGCGCATCAAT
1B37	TTTTGGTGCACGTTCGCT

Table S4. Boundaries of initial parameter values that were used to fit the DNA-binding model to experimental SPR data.

	Cre		LiCre	
**Parameter**	**Lower bound**	**Upper bound**	**Lower bound**	**Upper bound**
α	1	2.5	1	2.5
$RUmax_h$	36	44	54	62
$RUmax_f$	24	30	36	44
k_t	0.08	1.1	0.08	1.1
k_1	-3	2	-3	2
k_{-1}	-3	2	-3	2
k_2	-4	2	-4	2
k_{-2}	-4	0	-4	0

Biology Open • Supplementary information

Table S5. Experimental data sets quantifying LiCre efficiency in yeast cells.

ID	Date	Strain	DMX Device	Intensity (mW/ cm^2)	Duration (min)	Regime	Temp. (°C)	Source	Related figures
1	2020-11	GY2214	Spot	35	60	Period: 10 s or 2 min<br>Duty: 0, 1, 5, 10, 50 or 100%	Room Temp.	Duplus-Bottin 2021	5e
2	2020-09&10	GY2214	Spot	1.5	60	Period: 2 min<br>Duty: 0, 5, 10, 50, 75 or 100%	Room Temp.	Duplus-Bottin 2021	5e
3	2021-10	GY2517	Box	35	7,20,60, or 180	Period: 10 s<br>Duty: 10%	30	This work	4h, 5f
4	2022-03-14	GY2517	Box	35	20 or 60	Period: 20 s<br>Duty: 10%	30	This work	4h, 5f
5	2022-03-21	GY2517	Box	35	7, 20 or 60	Period: 5 s<br>Duty: 10%	30	This work	4h, 5f
6	2022-04-04	GY2517	Box	35	7, 20 or 60	Period: 60 s<br>Duty: 10%	30	This work	4h, 5f
7	2022-04-12	GY2517	Box	35	7, 20 or 60	Period: 60 s<br>Duty: 33.3%	30	This work	5f
8	2019-07-11&12	GY2214	Spot	35	10, 20, 30, 60, 90, 180, 240	Period: 10 s<br>Duty: 5%	Room Temp.	This work	4e, 5d
9	2023-05-03	GY2753	Spot	various	60	Period: 7 s<br>Duty: 28.6%	30	This work	4i (red), 5g
10	2020-07-03	GY2214	Spot	35	60	Period: 10 s<br>Duty: 5%	20, 25, 30, 37	This work	4g
11	2024-04-12	GY2753	Spot	various	60	Period: 7 s<br>Duty: 28.6%	30	This work	4i (black)
12	2024-10-18	GY2517	Box	35	60	Period: 10 s<br>Duty: 10%	30	This work	4c, 4d, 6a
13	2025-01-17	GY2517	Box	35	7,20,60	Period: 10 s or 60 s<br>Duty: 0, 10, 20 or 50%	30	This work	6b
14	2025-11-26	GY2214 & GY2517	Box	35	60	Period: 10 s<br>Duty: 10%	30	This work	4f

Table S6. Assay evaluating frequency of plasmid loss

Culture	Dilution	CFUs on selective SD-W plates	CFUs on non-selective SD plates	Total CFUs on selective	Total CFUs on non-selective	Loss in this culture (%)	Loss (%)
A	A1	192	216	972	1083	10.25	21.45
		198	226				
		237	233				
	A2	115	148				
		114	125				
		116	135				
B	B1	226	293	859	1190	27.82	
		174	218				
		182	271				
	B2	92	145				
		91	144				
		94	119				
C	C1	198	271	889	1206	26.29	
		180	234				
		200	298				
	C2	101	150				
		112	122				
		98	131				

Table S7. Best-fit model parameters using dataset Nb 13 only. The model was fitted on LiCreWT data and on LiCreT418S data separately.

Parameter	Data set specificity	x = 1	x = 2	x = 3	x = 4
k_{OFF} (10^{-3} s^{-1})	LiCre-WT	79.4	39.7	19.9	8.9
k_{OFF} (10^{-3} s^{-1})	LiCre-T418S	100	62.9	39.7	17.7
R_0 (10^{-4} s^{-1})	LiCre-WT	2.2	2.2	2.2	2.5
R_0 (10^{-4} s^{-1})	LiCre-T418S	2.2	2.5	2.8	3.2
Fit score[a]	LiCre-WT	0.097	0.097	0.099	0.102
Fit score[a]	LiCre-T418S	0.097	0.094	0.091	0.088

(a) Chi-square-like score quantifying distance to observations. The lower this score, the better the fit (see Methods).
